# Valedictory

**Published:** 1857-06

**Authors:** D. L. McGugin


### Valedictory.

The necessity which constrains us to surrender the editorial charge of the *Journal* is attended with unfeigned regret and sorrow. This does not arise, however, from any apprehensions as to its welfare in the future; on the contrary, although it has passed into the hands of others, its interests will be enhanced and its character for usefulness fully sustained; but because a kindly relation, which has subsisted between ourselves and our readers for the term of three years, has engendered friendships and familiarities which require a much colder and more frigid nature than our's to set aside without betraying at least some emotion, however sordid we may be regarded by the world.

When we projected this *Journal* no publication of this kind existed in the state. The profession felt and deplored the want, and many sent us up the most urgent appeals that it might be supplied. We hesitated because inexperienced in journalism, and we feared a failure in attempting to meet the demands of the profession. Nor did we know how far it could be sustained; for should we make the effort we would be ambitious to succeed. Since then it has grown steadily and surely, and now *it is one of the permanent journals of the country*. Patient labor, anxious care, continued exertion, and unwearied attention, were required to direct the tottering steps of its feeble infancy; but as this period has been safely passed, it can now boast of the health and strength of vigorous youth, and quite consistently with that part of life's history, it has its aspirations and hopes of a favorable future.

When we embarked in the enterprise we had very many difficulties to encounter and some opposition to overcome. This last was from a source so dishonorable and contemptible that it died from the malignancy, the inherent poison, rankling in its own organization, and dying, emitted a specimen or two of that phosphorescent light which usually attends the putrescency of such corrupt material. With two or three such flickerings, it was gone; yet we pursued our onward way, neither looking to the right nor the left, pressing forward with honest purposes and motives.

We have aimed to cull the best fruits which have ripened from time to time on the expanding tree of medical knowledge, and to present them to our readers. We have also given with pleasure the original productions of our contributors, and we have been gratified to find that they have generally been appreciated by our brethren of the press, who have transferred very many of them to their columns. We have, without hesitation or reserve, let loose our "war-dogs" upon empiricism and all infidel-*isms*, and other *schisms* in medicine.

But we are admonished with the shortness of time and the brevity of our space. We will only add here that in the third volume we were most efficiently aided by our talented and classical friend, Prof. Jno. R. Allen, M. D., than whom we never met a more chaste and classical writer. This we say in justice to one so deserving, who is now absent from home.

To one and all of our former friends we say FAREWELL, which, so far as editorial relations are involved, is, we think, *final*. We never expect again to enter upon the discharge of these duties. Our years are rolling away, and with them in the rapid transit of time, more or less of our physical vigor and vital stamina. We are most severely and painfully admonished that we must not assume too much labor, and we have acted in accordance with the reasonable suggestion.

In closing—be entreated to stand by your journal as you would stand by your profession, and both shall be increasing objects of solicitude and devotion, paramount to almost all other considerations.

D. L. McGugin.

